# Effect of adjuvant cisternostomy on the prognosis of patients with severe traumatic brain injury: A single center’s retrospective study

**DOI:** 10.1097/MD.0000000000041699

**Published:** 2025-03-07

**Authors:** Peng Chen, Wenxin Zheng, Wei Wang, Daokun Shu, Dongdong Xu, Qiquan Zhang, Bin Wang, Yi Zhuo, Raorao Yuan, Shiqi Cheng

**Affiliations:** a Department of Neurosurgery, The Second Affiliated Hospital of Nanchang University, Nanchang, China; b Department of Neurosurgery, Faculty of Medicine of Nanchang University, Nanchang, China; c Department of Neurosurgery, Leping Municipal People’s Hospital, Leping, China; d Department of Neurosurgery, Pengze County People’s Hospital, Jiujiang, China; e Department of Neurosurgery, Guangchang County People’s Hospital, Fuzhou, China.

**Keywords:** cisternostomy, decompressive craniectomy, IL-1β, nerve fiber layer, severe traumatic brain injury

## Abstract

This study evaluates the efficacy of adjuvant cisternostomy (AC) versus decompressive craniectomy (DC) in managing severe traumatic brain injury (sTBI). A single-center retrospective analysis of consecutive sTBI patients treated with AC or DC alone (2018–2019) revealed that the AC group exhibited significantly lower intracranial pressure and higher Glasgow Coma Scale scores compared with the DC group (*P* < .05). Cerebrospinal fluid and serum analyses demonstrated reduced interleukin-1β and nerve fiber layer levels in the AC group. In addition, the AC group required lower mannitol dosages and showed fewer postoperative complications. Six-month follow-up indicated a statistically higher rate of good prognosis in the AC group (*P* < .05). These findings suggest that AC is superior to DC in reducing intracranial pressure, mitigating neurological damage, and improving clinical outcomes in sTBI patients.

## 1. Introduction

Traumatic brain injury (TBI) is a common disease that endangers human health. According to the World Health Organization, the incidence rate of TBI is on a steady rise, and the main morbidity population is the young adults group. Severe traumatic brain injury (sTBI) accounts for about 10% of TBI. Its rising mortality (over 20%) and disability rate (over 50%) are one of the main causes of death and disability in the world.^[1,2]^ The pathogenesis of sTBI can be divided into the following 2 aspects: the primary injury caused by direct physical impact and the secondary injury resulting from brain ischemia and hypoxia, brain edema and increased intracranial pressure, metabolism, oxidative stress, excitotoxic reaction, inflammatory cascade reaction, and so on.^[3–5]^ Meningeal lymphatic vessels, a structure discovered in recent years, are responsible for removing macromolecules, cellular debris, and immune cells from the brain, thereby maintaining intracerebral homeostasis. Recent studies, such as the one conducted by Bolte et al^[6]^ using a mouse model, have shown that impaired lymphatic drainage following TBI leads to an upregulation of lymphatic vessel endothelial hyaluronan receptor 1 (LYVE-1), exacerbating neuroinflammation (indicated by increased levels of glial fibrillary acidic protein and ionized calcium-binding adapter molecule 1), and cognitive dysfunction.

How to effectively control intracranial pressure (ICP) is important for treating sTBI.^[7]^ Surgical intervention occupies an important position in sTBI treatment, especially for patients with ineffective drug treatment.^[8]^ In the past few decades, decompressive craniectomy (DC), a world-recognized classic and standard surgical method for the treatment of intractable intracranial hypertension caused by cerebral edema, has been written into the neurosurgery guidelines for the treatment of severe head injury.^[9]^ This operation can reduce the incidence and mortality of cerebral hernia, but there are still many complications.^[10,11]^ Recent reports indicate that patients who survive 3 months after DC often require cranioplasty, which can increase patient discomfort and economic burden.^[12,13]^

Adjuvant cisternostomy (AC) is a new technique for treating sTBI. Compared with DC, AC has been preliminarily proven to reduce the incidence of complications, disability, and mortality of patients in sTBI and improve the clinical outcome and prognosis.^[14,15]^ The principle of this operation is to recognize the important role of the perivascular pathway in the Virchow–Robin space in cerebrospinal fluid circulation.^[16]^ Opening the skull base brain cisterns (such as the chiasmatic cistern, carotid cistern, and pontine cistern) to release and drain cerebrospinal fluid can reverse “displaced cerebral edema,” thereby reducing ICP.^[14,15]^ And continuous drainage of cerebrospinal fluid after an operation could scavenge metabolites such as oxygen free radicals and histamine and reduce a series of secondary brain damage caused by cerebral ischemia and hypoxia.^[17,18]^

Neurofilament light chain is involved in the formation of the neuronal cytoskeleton and plays a key role in maintaining cell morphology and myelin axon regeneration. Hajiaghamemar et al^[19]^ have confirmed that in the context of TBI, neurons, astrocytes, and blood vessels have been damaged, causing the release of specific protein components such as nerve fiber layer (NFL), neurofilament heavy chain, and glial fibrillary acidic protein. At the same time, studies have shown that processes such as brain tissue necrosis and repair after trauma involve the massive release of IL-1β, TNF-α, and other inflammatory factors.^[20–22]^ These biomolecules play an important role in the secondary injury of TBI, especially in the formation of brain edema after TBI, and may be used as biomarkers to evaluate the severity of trauma and even the prognosis.^[23]^ Therefore, the role of these biomarkers (IL-1β and NFL) in the treatment of sTBI deserves further exploration.

This study aimed to compare the prognostic differences between patients in the DC and AC groups by detecting classical TBI cerebrospinal fluid markers and common clinical indicators. Initially, we compared the AC and DC groups by monitoring ICP and calculating Glasgow Coma Scale (GCS) scores, assessing the correlation between these 2 metrics. We also examined differences in the expression of IL-1β and NFL by analyzing cerebrospinal fluid specimens from TBI patients. Finally, we analyzed the incidence of postoperative complications and Extended Glasgow Outcome Scale (GOSE) scores in both groups.

## 2. Methods

### 2.1. Patient group

The Research Ethics Committee of The Second Affiliated Hospital of Nanchang University approved this single-center retrospective cohort study. Based on the inclusion and exclusion criteria, a total of 43 surgical patients with sTBI were selected. These included 19 patients in the standard DC group and 24 patients in the cisternal fistula group (Table 1). Additionally, cerebrospinal fluid and serum samples were collected from 20 healthy volunteers who had no abnormal physical examination results, signed informed consent, and met medical ethics requirements. The levels of IL-1β and NFL in the cerebrospinal fluid and serum of the 2 groups of surgical patients were then measured.

**Table 1:** General information of the cisternostomy group and the DC group.

General characteristics	Cisternostomy group (n = 19)	DC group (n = 24)	*P* value
Gender (male)	36.84%	33.33%	.811
Age (years)	56.58 ± 12.26	56.04 ± 11.89	.803
Injury method			.759
Traffic accident Injury	10 (52.63%)	11 (45.83%)	
Fall from height	6 (31.58%)	7 (29.17%)	
Hit injury	3 (15.79%)	6 (25.00%)	
Pupil changes			.546
Normal	14 (73.68%)	16 (66.67%)	
Simple dilated pupils	5 (26.32%)	8 (33.33%)	
Image diagnosis			.785
Brain contusion with bleeding	10 (36.84%)	13 (29.17%)	
Traumatic subdural hematoma	2 (10.53%)	4 (16.66%)	
Mixed injury	7 (52.63%)	7 (54.17%)	
Degree of shift of brain midline	0.83 ± 0.18	0.91 ± 0.15	.174
Time from injury to hospital admission (hour)	8.81 ± 4.03	8.30 ± 3.12	.646
Time from injury to operation (hour)	11.72 ± 3.95	12.02 ± 3.41	.782
Operation time (hour)	4.36 ± 0.98	4.04 ± 0.85	.271

DC = decompressive craniectomy.

### 2.2. Postoperative patient treatment and management

#### 2.2.1. ICP monitoring

The ICP-monitoring probe (Codman, Johnson & Johnson, Raynham) was implanted into the contralateral ventricle with the most serious damage. For patients undergoing craniotomy due to mass effect lesions, the contralateral ventricle has the most serious damage. All working ends were connected to the ICP monitor and ICP values were recorded. The head computed tomography scan was completed within 6 hours after surgery to verify the placement of the ICP-monitoring probe.

#### 2.2.2. Comprehensive preoperative management of sTBI

In this study, all sTBI patients admitted to our hospital’s neurosurgery center received standardized preoperative management following the fourth edition of the US “Guidelines for the Treatment of Severe Head Injuries.” All patients were given sedation and mechanical ventilation to maintain the arterial partial pressure of oxygen >60 mm Hg, the arterial partial pressure of carbon dioxide between 35 and 40 mm Hg, and the cerebral perfusion pressure between 60 and 70 mm Hg, which ensure the stability of blood vessel osmotic pressure and internal environment. Metabolic control included maintaining normal blood sugar, body temperature, and hormone levels.

### 2.3. Surgical treatment

#### 2.3.1. Standard decompression group

For all patients in the DC group, the surgeon performed unilateral or bilateral frontotemporal standard decompression to remove any obvious hematomas according to the needs of the disease and surgical experience. In this series, a craniotomy was included, with the medial margin about 1 cm from the midline and at least 12 cm in anteroposterior diameter to the bottom of the middle cranial fossa. The dura mater was incised in a stellate manner. After the operation, the artificial dura mater was expanded and sutured to restore the tightness of the cranial cavity to prevent postoperative cerebrospinal fluid leakage.

#### 2.3.2. AC group

Since January 2016, our center has carried out cisternostomy. The surgical procedure has been described earlier, the basal basin has been opened to atmospheric pressure and a catheter has been placed in the chamber. Through transpersonal approach or enlarged pterional approach craniotomy, the sphenoid crest is bitten off and flattened, reaching the supraorbital fissure, above the supraorbital ridge, and the dura has been cut parallel to the frontal floor. The cistern has been opened through the frontal floor to sharply separate the carotid cistern. The arachnoid membrane could be separated from the oculomotor nerve, and the Liliequist membrane could be separated from the optic chiasm and the carotid space (second space). Then in the prep ontic cistern, the brain cistern was opened through the lateral fissure, and the optic chiasma was found according to the intersection of the olfactory tract. The disc wing and optic chiasm have been opened. The pontine cistern was subsequently exposed. A standard ventricular drainage tube was placed in the cisternal ventricle to drain the cerebrospinal fluid. Subsequently, the dura mater was meticulously sutured, the skull was repositioned and secured using cranial bone connectors, and the scalp was sutured.

#### 2.3.3. Management of postoperative ICP

All sTBI patients in this study underwent ICP monitoring after surgery and stepped ICP treatment was adopted according to the dynamic changes of ICP. The cerebral perfusion pressure has been maintained between 60 and 70 mm Hg while controlling ICP ≤ 20 mm Hg. The first stage: controlled the body position to keep the head up at 30° and carried out appropriate sedation and analgesia treatment; the second stage: monitored the electrolyte and blood pressure and gave mannitol or hypertonic drugs according to the condition; the third stage: for refractory intracranial hypertension, barbiturate therapy is recommended, provided hemodynamic stability is maintained. It should be noted that mild hypothermia can reduce the oxygen consumption of brain tissue, keeping the body temperature of sTBI patients between 30 and 35°C. If the aforementioned treatments fail, a second craniotomy is required.

### 2.4. Biomarker detection

#### 2.4.1. Collection, processing, preservation, and inspection of cerebrospinal fluid

Cerebrospinal fluid samples were collected from the ventricular drainage catheter during and after the operation (5–10 mL/time) and added to a sterile test tube. Then the collected cerebrospinal fluid was centrifuged at 2500 rpm for 10 minutes at 4°C and the supernatant was captured and frozen at −20°C for later use. According to the needs of the experiment, an appropriate number of samples was taken for enzyme-linked immunosorbent assay determination.

#### 2.4.2. Collection, processing, preservation, and inspection of serum

About 3 mL of fresh blood was collected with an ordinary vacuum blood collection tube or coagulation tube and added to a sterile test tube. Then the collected blood was centrifuged at 3000 rpm for 20 minutes at 4°C, and the supernatant was captured and frozen at −20°C for a later test. The enzyme-linked immunosorbent assay double antibody sandwich method was used to analyze the levels of IL-1β (ab46052 [Abcam plc, Cambridge, UK], sensitivity < 6.5 pg/mL) and NFL (E-EL-H1888c [Elabscience Biotechnology Co., Ltd., Houston], sensitivity < 10 pg/mL) in serum and cerebrospinal fluid.

#### 2.4.3. Data collection

The collection of clinical data included the patient demographic data, changes in GCS scores, associated injuries, and duration of surgery, as well as the retention of serum and cerebrospinal fluid samples. The early clinical outcome indicators of the study included postoperative ventilator time spent, intensive care unit hospital stay, GCS at intensive care unit discharge, the incidence of postoperative complications, secondary bone removal rate, and the number of survival/death 2 weeks after surgery. The long-term clinical outcome (6 months after surgery) was evaluated using the GOSE.

### 2.5. Statistical methods

All data were statistically analyzed using SPSS 21.0 software (IBM Corporation, Armonk). Categorical variables are presented as composition ratios or frequencies (%). To compare differences between the 2 groups, chi-square tests or Fisher exact tests were used for categorical variables, and Student *t* test or the Mann–Whitney *U* test was employed for quantitative variables. The data are expressed as mean ± standard deviation, with *P* < .05 considered statistically significant. The correlation was analyzed using Pearson correlation analysis. Graphics were created using GraphPad Prism 8.0 (GraphPad Software, Inc., San Diego) series software and R version 4.0.3 (R Project for Statistical Computing, R Foundation, Vienna, Austria).

## 3. Results

### 3.1. Postoperative ICP and GCS scores in the DC group and AC group

**Figure 1.**
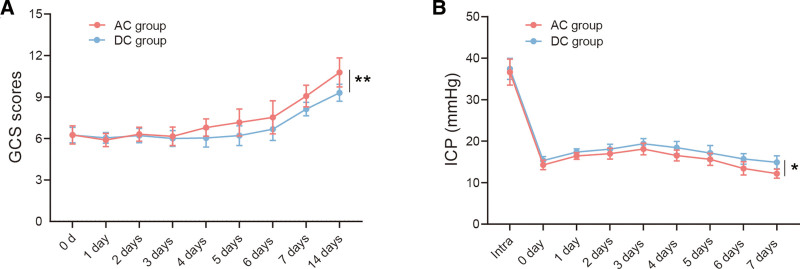
ICP and GCS scores in the AC group and the DC group on different time points. (A) Line chart of ICP in the AC group and the DC group before and after operation; (B) Line chart of GCS scores in the AC group and the DC group before and after the operation. *P < .05. AC = adjuvant cisternostomy, DC = decompressive craniectomy, GCS = Glasgow Coma Scale, ICP = intracranial pressure.

After the surgical operation, the ICP in both groups reduced significantly and could be kept within the normal range during the first week. Besides, on the 6th and 7th days after the operation, the ICP in the AC group was significantly lower than that in the DC group (*P* < .05) (Fig. 1A). From the 7th day to the 14th day, the GCS scores of the AC group were significantly higher than that of the DC group (*P* < .05) (Fig. 1B).

**Figure 2.**
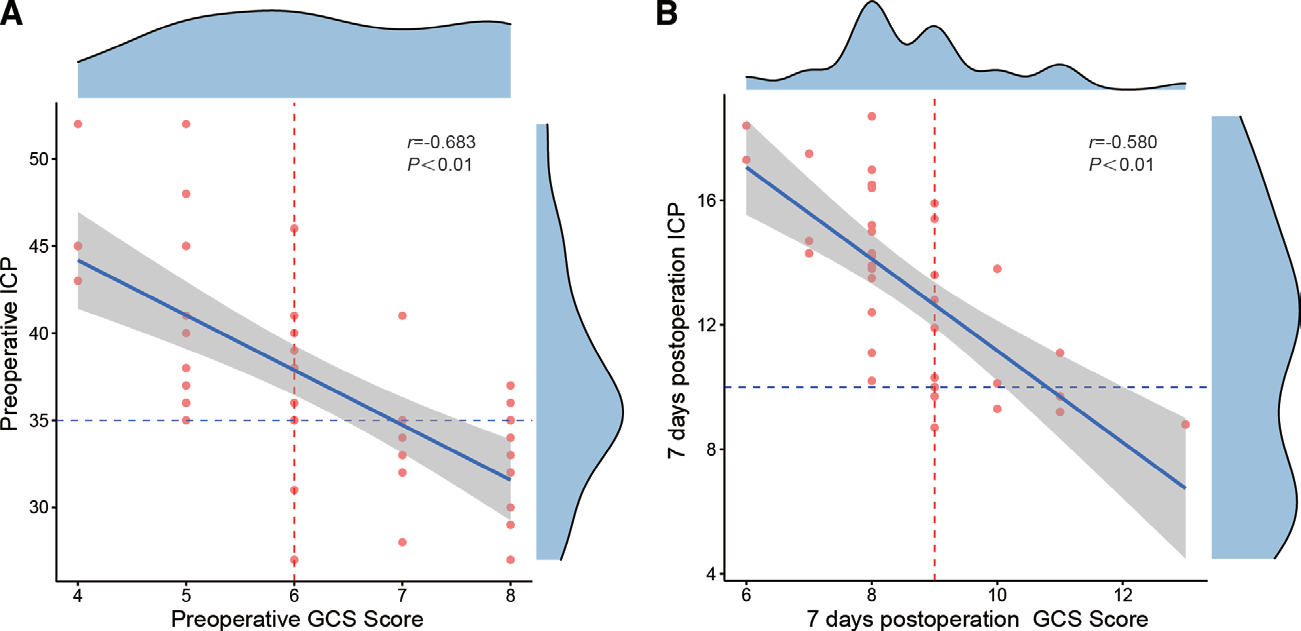
Correlation between GCS scores and ICP in different periods. (A) Relevance between preoperative GCS scores and ICP; (B) Relevance between GCS scores and ICP in 7 days after the operation. GCS = Glasgow Coma Scale, ICP = intracranial pressure.

To further explore the relationships between GCS scores and ICP, we performed correlation analysis and found that there was a negative correlation between these 2 measurements before and after the operation in patients (*r* = −0.683 and *r* = −0.580, respectively, *P* < .01) (Fig. 2).

### 3.2. The levels of IL-1β and NFL in cerebrospinal fluid and serum in the AC group and the DC group

**Figure 3.**
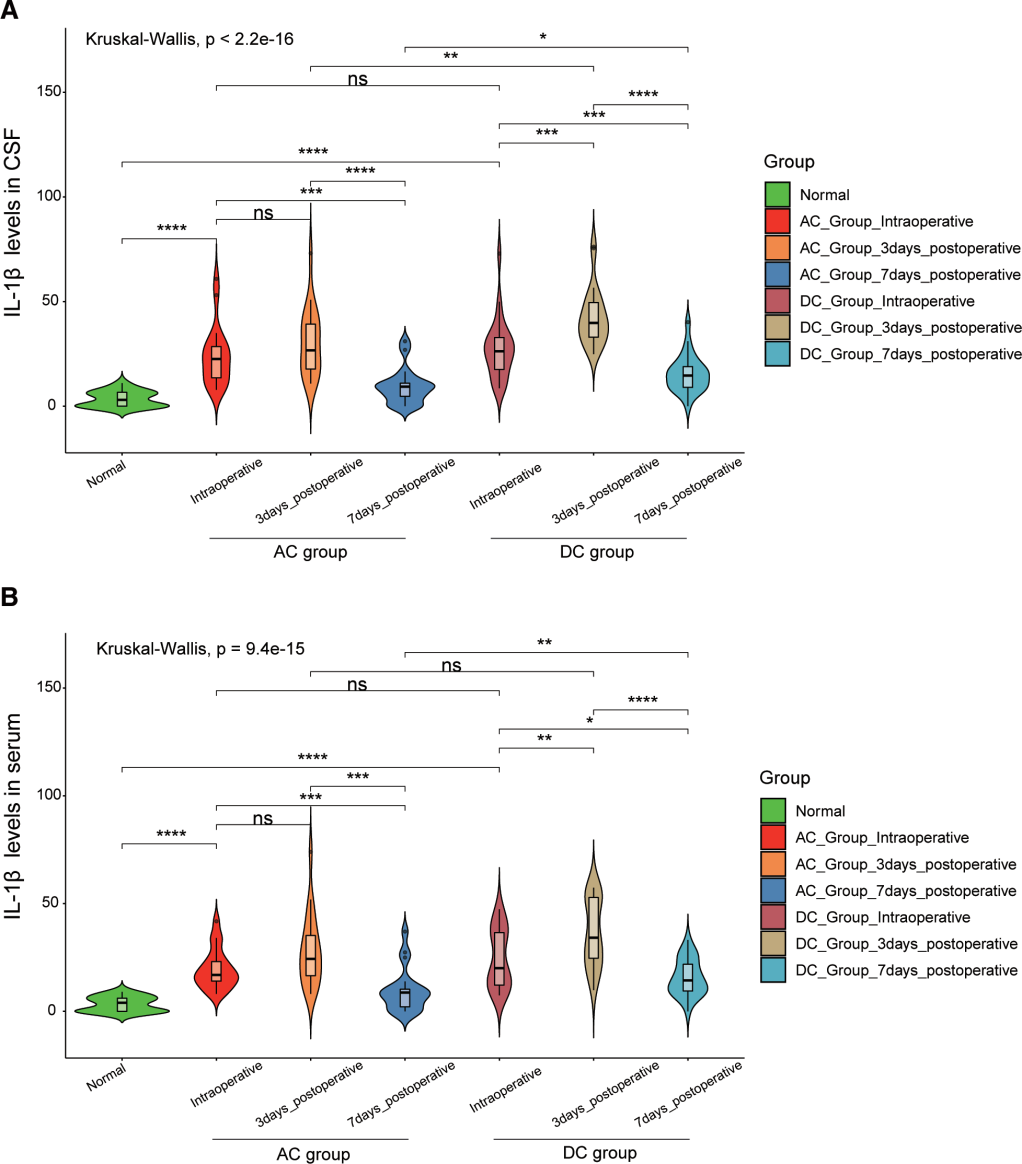
The level of IL-1β in cerebrospinal fluid and serum in the AC group and the DC group. (A) The level of IL-1β in cerebrospinal fluid in the AC and DC groups; (B) The level of IL-1β in serum in the AC and DC groups. *P < .05, **P < .01, ***P < .001, ns means has no statistical significance. AC = adjuvant cisternostomy, DC = decompressive craniectomy, IL-1β = interleukin-1β.

**Figure 4.**
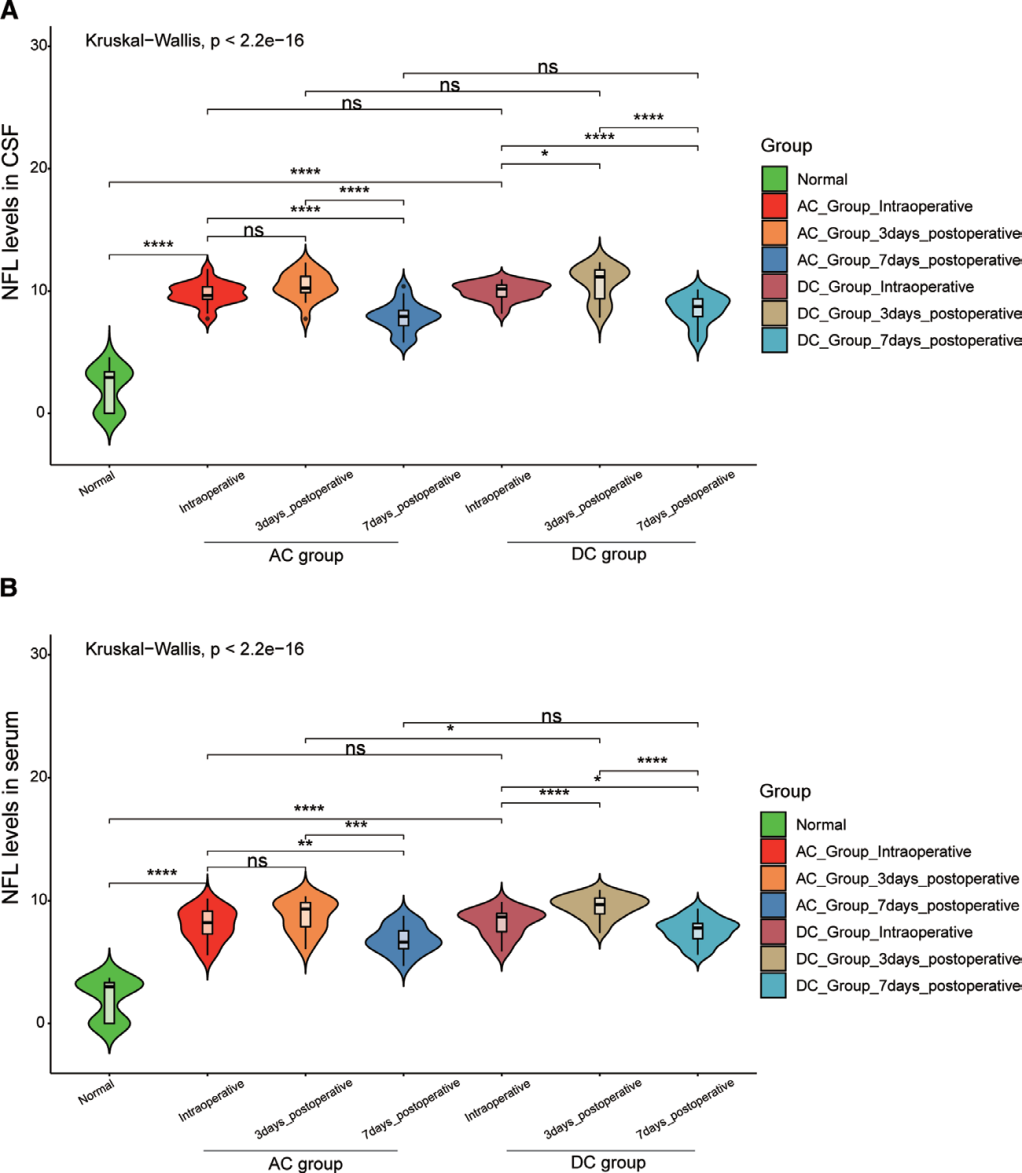
The level of NFL in cerebrospinal fluid and serum in the AC group and DC group. (A) The level of NFL in cerebrospinal fluid in the AC and DC groups; (B) The level of NFL in serum in the AC and DC groups. *P < .05, **P < .01, ***P < .001, ns means has no statistical significance. AC = adjuvant cisternostomy, DC = decompressive craniectomy, NFL = nerve fiber layer.

**Figure 5.**
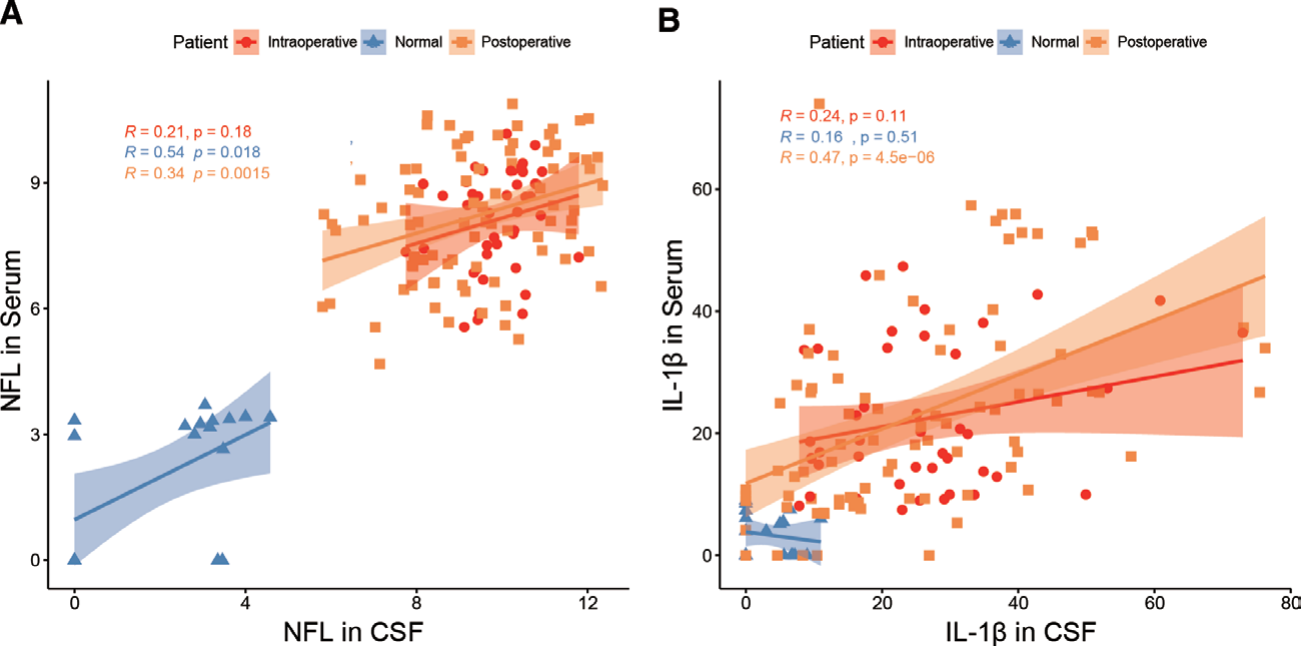
Correlation analysis of IL-1 and NFL in serum and cerebrospinal fluid. (A) Correlation analysis of IL-1 in cerebrospinal fluid and serum at different periods. (B) Correlation analysis of NFL in cerebrospinal fluid and serum at different periods. IL-1 = interleukin-1, NFL = nerve fiber layer.

The levels of IL-1β and NFL in cerebrospinal fluid and serum in the AC group were lower than those in the DC group. On the 3rd and 7th postoperative days, IL-1β levels in both cerebrospinal fluid and serum were consistently lower in the AC group compared to the DC group, and that this difference was statistically significant (*P* < .05 for cerebrospinal fluid and *P* < .01 for serum) (Fig. 3A, B). Similarly, the NFL in cerebrospinal fluid and serum of the AC group was lower than that of the DC group (*P* < .01) (Fig. 4A, B). We also analyzed the correlation between IL-1 and NFL expression in brain fluid and serum (Fig. 5A, B). The results suggest a positive correlation between the expression levels of IL1β and NFL in both serum and cerebrospinal fluid, indicating that IL1β and NFL markers can be utilized for prognostic predictions in sTBI patients through a less invasive blood collection method.

### 3.3. Postoperative complications and the Glasgow extended prognostic scores in the DC group and the AC group

**Figure 6.**
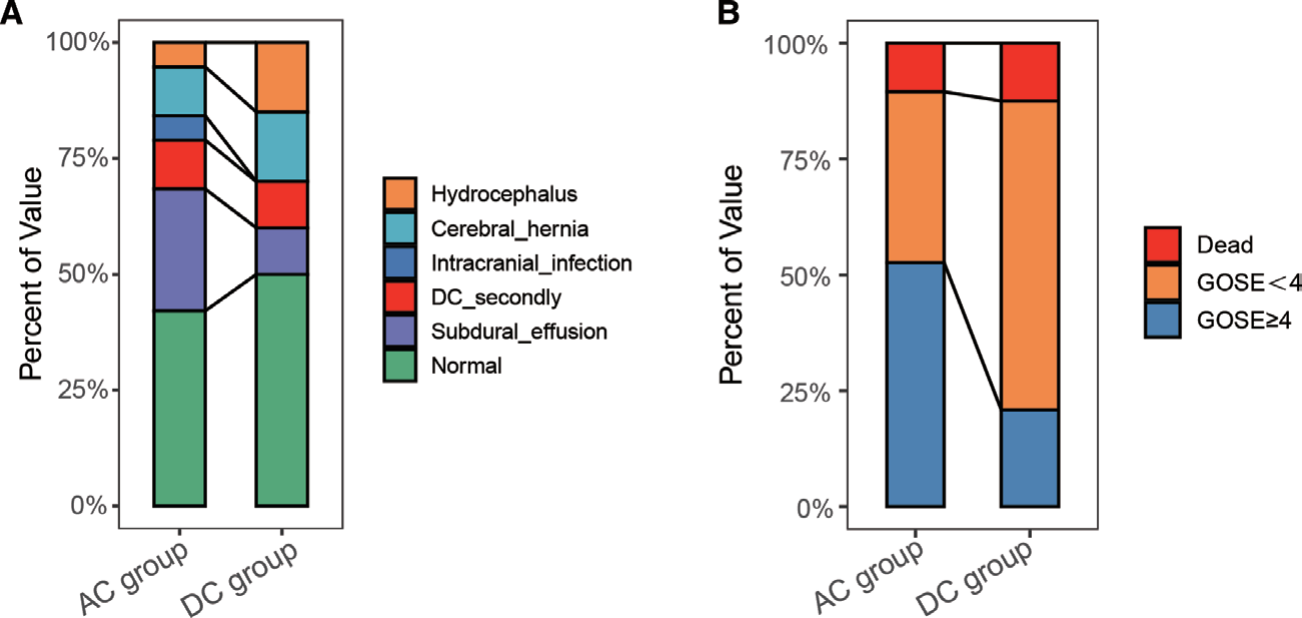
Prognosis of patients in the DC and AC groups. (A) The postoperative complications of patients in the AC and DC groups were analyzed. (B) The distribution of GOSE scores among different groups. AC = adjuvant cisternostomy, DC = decompressive craniectomy, GOSE = Extended Glasgow Outcome Scale.

In postoperative management, the AC group has a mostly lower incidence of complications after operation compared with the DC group (Fig. 6A), such as second bone flap removal, subdural effusion, cerebral hernia, and posttraumatic hydrocephalus after 6 months after surgery.

According to a 6-month follow-up by GOSE evaluation of the 2 groups, the good prognosis rate of the AC group (52.63%) was higher than that of the DC group (20.83%) (*P* < .05) (Table 1). Statistics of postoperative complications showed that only 1 case (5.26%) had traumatic hydrocephalus in the AC group, but there were 3 cases (12.50%) in the DC group. Subdural effusion occurred in 2 cases (10.53%) in the AC group, while 5 cases in DC group (20.83%); The incidence of postoperative cerebral hernia in AC group (2 cases,10.60%) was lower than that in DC group (3 cases, 12.50%) (*P* = .841) (Fig. 6B and Table 2).

**Table 2:** Extended Glasgow Outcome Scale assess table.

Prognosis	Cisternostomy group (n = 19)	DC group (n = 24)	*P* value
Dead/live patient after postoperative 2 weeks (%)	2/17 (10.53%)	3/21 (12.50%)	.841
Patient status after 6 months postoperative (%)			
GOSE 1	2 (10.53%)	3 (12.50%)	
GOSE 2	2 (10.53%)	5 (20.83%)	
GOSE 3	2 (10.53%)	5 (20.83%)	
GOSE 4	3 (15.79%)	6 (25.00%)	
GOSE 5	6 (31.58%)	2 (8.33%)	
GOSE 6	3 (15.8%)	2 (8.33%)	
GOSE 7	1 (5.26%)	1 (0.04%)	
GOSE 8	0 (0.00%)	0 (0.00%)	
GOSE > 4	10 (52.63%)	5 (20.83%)	
GOSE ≤ 4	9 (47.37%)	19 (79.17%)	
Good prognosis rate (GOSE > 4)	10 (52.63%)	5 (20.83%)	.03

DC = decompressive craniectomy, GOSE = Extended Glasgow Outcome Scale.

## 4. Discussion

Even though great progress has been made in the surgical technique of sTBI, the clinical outcome of patients with this disease is still not ideal due to the lack of effective surgical strategies and accurate prognostic signatures for real-time monitoring of sTBI. AC is a newly-applied technique for sTBI treatment. Many studies have shown that the process of brain microcirculation disorder, brain tissue necrosis, damaged brain tissue repair, and other processes would cause a large amount of release of inflammatory factors such as IL-6, IL-1β, NFL, and TNF-α. However, the prognostic performance of cisternostomy, IL-1β, and NFL in sTBI has not been explored previously.

ICP is one of the most important indexes to evaluate the therapeutic effect. In this study, ICP data of the AC group and the DC group were analyzed at different time points. It was found that compared with DC group, the overall ICP level of the AC group was lower than that of the DC group, which indicated that the AC benefited better than DC. Giammattei et al^[24]^ also found that patients in the cisternostomy group have significantly lower average postsurgical ICP values, higher GCS scores, and require less osmotic treatments compared with those treated with DC alone. From the 7th day to the 14th day, the GCS scores of the AC group were significantly higher than that of the DC group (*P* < .05), and we also found that there was a negative correlation between GCS scores and ICP before and after operation (*r* = −0.683 and *r* = −0.580, respectively, *P* < .01). These results demonstrated that a high GCS score was accompanied by a decrease of ICP, which further proved that AC was beneficial to the prognosis of sTBI patients.

According to the related published literature, IL-1β and NFL were closely related to craniocerebral injuries. Recently, Gatson et al^[25]^ and Siman et al^[26–28]^ found that serum NFL was a potential predictor of injury severity within 72 hours after TBI, and the levels of phosphorylated neurofilament heavy chain in the blood and cerebrospinal fluid during the acute and chronic phases of secondary TBI have correlated with the severity of mild, moderate, and sTBI. On the 3rd and 7th day after the operation, compared with the DC group, IL-1β and NFL in cerebrospinal fluid and serum of the AC group were lower than that of the DC group. All the evidence proved that combined detection and monitoring of IL-1β and NFL in serum and CSF will contribute to patients with sTBI. Reducing IL-1β and NFL could effectively reduce brain inflammatory response and protect normal brain function.

Also, the used dosage of mannitol and incidence of complications in the AC group was lower than that in the DC group. Meanwhile, GCS scores and the rate of good prognosis were higher in the AC group compared with the DC group. Although cisternostomy was indeed advantageous in the treatment of sTBI, some of its risks could not be ignored. Kurland et al^[29]^ conducted a systematic retrospective analysis of the relevant literature and found that the incidence of traumatic hydrocephalus after DC was about 15%, and the incidence of subdural effusion was 27.4%. Dimitriou et al^[30]^ found the risk of intracranial infection in ICP-monitoring probe implantation increased from 5 days after the operation, and its peak was 5 to 10 days after probe implantation. In this study, the incidence of intracranial infection in the AC group (5.26%) was slightly higher than that in the DC group (0.00%). Therefore, we should pay attention to the management of postoperative drainage tubes to reduce the risk of postoperative cerebral hernia, secondary operation, and intracranial infection. The above results all demonstrated that compared with the DC group, the AC group showed better ICP control, less postoperative mannitol administration, and a better prognosis. At the same time, the monitoring of IL-1β and NFL during and after the operation could provide more reliable information to indicate the severity of the disease, thereby guiding the timely and precise clinical intervention.

Overall, our retrospective analyses showed that compared with the DC group, sTBI patients who underwent an AC had a better clinical outcome, their ICP control and GCS scores presented a favorable normal range on postoperative 6 to 14 days. Besides, our data indicated that there was a negative correlation between GCS scores and ICP levels before and after the operation. Moreover, the levels of IL-1β and NFL in cerebrospinal fluid and serum in the ACC group were lower than those in the DC group, which are considered to have something to do that might be conducive to a better prognosis. Besides, AC had a marked effect on releasing the reduction of subsequently used mannitol dosage and postoperative complications, parameters that held a certain influence on the clinical outcome of the patients.

## 5. Conclusion

AC is a strong performer in the treatment of sTBI compared with DC. However, its challenge is to open a cistern in the swelling of brain tissue after sTBI, which requires higher surgical techniques. In addition, monitoring the changes of IL-1β and NFL levels in cerebrospinal fluid and serum of sTBI patients has great clinical application value for judging the severity of brain injury, the prognosis of the patient, and evaluating the curative effect.

Although this study has achieved good results, due to its small sample size, short postoperative follow-up time, and the form of single-center research (single-center retrospective case–control study), we still need a large number of multicenter clinical randomized double-blind studies to enrich and collect more data to improve persuasion.

## Author contributions

**Conceptualization:** Peng Chen, Daokun Shu, Bin Wang.

**Writing – original draft:** Peng Chen.

**Data curation:** Wenxin Zheng, Daokun Shu, Yi Zhuo.

**Investigation:** Wenxin Zheng, Qiquan Zhang.

**Formal analysis:** Wei Wang.

**Project administration:** Wei Wang, Dongdong Xu, Qiquan Zhang, Raorao Yuan.

**Methodology:** Dongdong Xu.

**Visualization:** Bin Wang.

**Resources:** Yi Zhuo.

**Supervision:** Raorao Yuan.

**Funding acquisition:** Shiqi Cheng.

**Writing – review & editing:** Shiqi Cheng.
